# Two thirds of the most disadvantaged Dalit population of Nepal still do not deliver in health facilities despite impressive success in maternal health

**DOI:** 10.1371/journal.pone.0217337

**Published:** 2019-06-03

**Authors:** Surendra Prasad Chaurasiya, Nilesh Kumar Pravana, Vishnu Khanal, Dhiraj Giri

**Affiliations:** 1 Maharajgunj Medical Campus, Institute of Medicine, Tribhuvan University, Kathmandu, Nepal; 2 Nepal Development Society, Bharatpur, Nepal; 3 Department of Natural Science, Kathmandu University, Kathmandu, Nepal; University of Ghana, GHANA

## Abstract

**Introduction:**

The gains in maternal and child health in Nepal was impressive in the last two decade but success was unevenly distributed. The Dalits of Nepal are the most disadvantaged caste group and have benefitted least from the advances in maternal health service. This study investigated the rate of and factors associated with the institutional delivery among the Dalit women of the Mahottari, Nepal.

**Materials and methods:**

A cross-sectional study was conducted during July-December 2014 using a structured questionnaire. A total of 328 mothers who had their childbirth within one year were interviewed. Descriptive statistics followed by binary and multivariable logistic regression analyses were computed to find the association of key variables with institutional delivery.

**Results:**

In this study, only 30% of the mother had institutional delivery. Fifty eight percent mothers had no any birth preparedness and complication readiness. Four or more antenatal visits (Adjusted Odds Ratio (AOR): 3.54, CI: 1.82–6.90), birth preparedness (AOR: 3.15, CI: 1.61–6.18), planned pregnancy (AOR: 2.63, CI: 1.37–5.06) and receiving advice from health staffs (AOR: 3.96, CI: 2.00–7.86) and mother's autonomy (AOR: 2.25, CI: 1.03–4.49) were associated with child birth at the health facility.

**Conclusion:**

This study indicated that birth preparedness, ANC visit frequency, planning of pregnancy, advice for institutional delivery and mother's autonomy were significantly associated with health facility delivery. Less than one-third mothers had institutional delivery and reasons were feeling of un-necessary, far distance, lack of transportation and associated cost; and birth preparedness is also low. Hence, promotion of birth preparedness, uptake of ANC service, proper counselling for institutional delivery, promoting women autonomy and strengthening women to have planned pregnancy were some recommendation to promote institutional delivery for such disadvantage community.

## Introduction

Institutional delivery and access to referral level facility are essential intervention in saving the lives of mothers and children in many developing countries [1]. Over the last decades, the maternal mortality ratio (MMR) was observed more than ten time higher in developing countries than in developed and almost all of the global maternal death (99%) occurred in developing countries, followed by sub-Saharan Africa region alone accounting for (179,000) and South Asia (69,000) [2]. Millennium Development Goals (MDG) has targeted to reduce MMR by three quarters between 1990 and 2015. Accordingly, the government of Nepal has committed to reducing MMR to 134 deaths per 100,000 live births; increase institutional delivery to 40% and increase delivery assisted by skilled birth attendants (SBA) to 60%,increase at least four Antenatal Care (ANC) visit to 80% [3, 4]. A recent report of the World Health Organization (WHO)shows that globally since 1990, MMR has been dropped by 44% but the world failed to meet the 75% reduction target[5].

As a result of Nepal’s committed efforts, the country has made a significant improvement in maternal health services in the past decade [4, 6, 7]. The overall MMR has been decreased (850 live birth per 100,000 in 1990 to 229 live birth per 100,000 in 2010) [3, 8]; delivery assisted by SBA has been increased by seven folds (7% in 1990 to 36% in 2011) [3]; health facility delivery has been increased (8% in 1996 to 35% in 2011); and four or more ANC visits has been increased (9% in 1996 to 50% in 2011) [6, 7].With such gains, the recently adopted ‘Sustainable Development Goals’ has globally summoned to reduce MMR to less than 70 per 100,000 live births by 2030 [5].

While the gains in maternal and child health in the country seem impressive, there is no uniformity. Nepal has a socially constructed caste-based hierarchical system in which the Brahmins/Chhetris are considered at the top (advantaged caste) and Dalits are at the lowest (disadvantaged caste) [9]. This system had an impact on the access of and utilization of health services by Dalit caste. Therefore, it is unsurprising that the gains in maternal health are also lowest for this group [3, 9]. For instance, MMR (national 229 versus Dalit 273), four or more ANC visit (Brahmin/Chhetri 64% versus Dalits 40%), institutional delivery (Brahmin/Chhetri 49% versus Dalit 26%) [9]. Further, a study showed increment in the difference between Brahmins/Chhetri and Dalits in ANC utilization from 10 to 19%; and institutional delivery from 5.7% in 1996 to 15.4% in 2006 [10]. These stats suggest that the gains were trickled more through much improvement in the upper caste groups, and the gains remain slower the in the Dalit caste.

Ecologically, Nepal has three regions, the northern Himalayan belt, middle hilly belt and the Southern plain (Terai). The central southern region of Nepal has a unique situation. This region is considered the food bank of the country where rice is grown as a major staple food. The plain terrain has an open border to India. Its plain terrain has more opportunity for development and also increased the likelihood of the use of health services in the areas. However, the national service utilization report shows that the statistics in this region is not as good as the hilly region. For instance, institutional delivery was 22% in Mahottari district while 45% at the national level in 2012/013 according to district health report and annual report respectively [11, 12].

Similarly, regional and geographical differences in health are well documented in Nepal, the situation of the Dalit caste group remain under-reported. To plan and implement public health programs focused to reduce maternal deaths, obstetric morbidities, and increase maternal health service utilization, well-designed studies are needed. Therefore, the objective of this study was to identify the rate of and factors associated with health facility-based childbirth among Dalit women who delivered their babies within one year in Mahottari district.

## Methods and materials

### Study setting

The study was conducted in the Mahottari district of central Nepal, which lies about 253 kilometres to the South-East to the capital of Nepal, Kathmandu. The southern part of district borders to the Bihar state of India. The district is divided into six electoral areas and each area consists of 11 to 14 village development committees (VDC—the smallest administrative unit in rural areas of Nepal) with a total VDC seventy seven including one municipality. Each VDC then is divided into nine wards which can be as small as a village. According to the National Population and Housing Census 2011, the total population of the district was 627,580 (male 311,016 and female 316,564) [13]. There are two district hospitals, three primary health care centres, 20 health posts, and 52 sub-health posts that provide primary health care services to its population [11]. The maternal health services provided through these public health facilities are free of cost according to the government of Nepal’s maternal health program guidelines. All the services related to maternal health services are collectively known as ‘Aama Program’ (means “Mother Program”). This program has four components: (a) the Safe Delivery Incentive Program, a cash incentive scheme initiated in July 2005, (b) free institutional delivery care, which was launched in mid-January 2009,(c) incentive to health worker for home delivery and (d) incentive to women for 4 ANC visits at the 4, 6, 8 and 9 months pregnancy following institutional delivery [12].The district was chosen purposively as this has unique representativeness of the Southern areas in the plain (Terai) region of Nepal.

### Study design and participants

**Fig 1:**
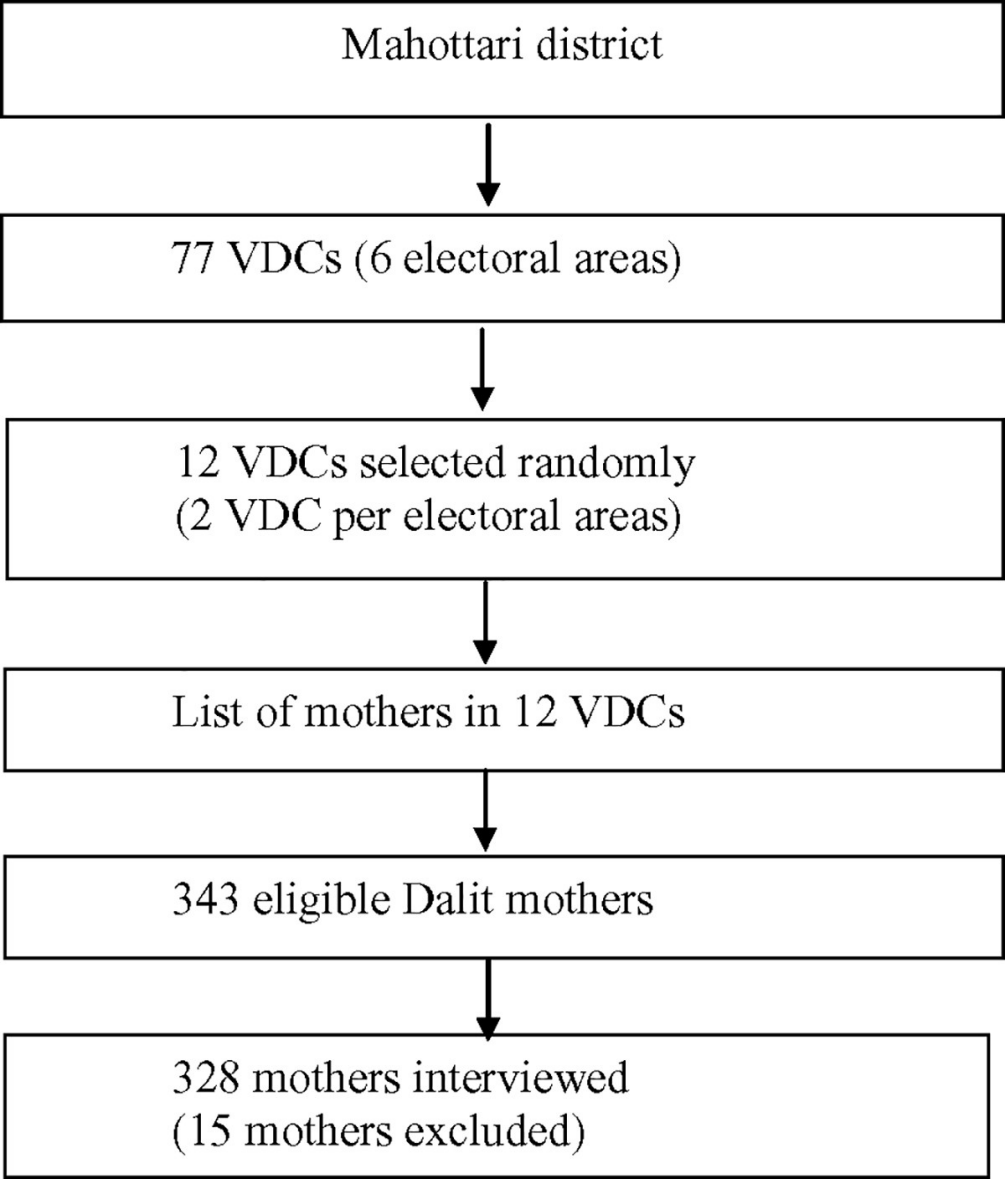
Sampling procedure.

This was a cross-sectional study conducted in Mahottari district during July-December, 2014. The mothers were included in the study if: (i) they had childbirth during the last one year, (ii) were local residents of the districts, (iii) were not migrated to the district after childbirth, and [14] belonged to Dalit ethnic group. A multistage simple random sampling technique was used. This district has six electoral areas and each area constitutes 11 to 14 VDCs (Fig 1). To ensure the representativeness, two VDCs were selected randomly selected from each electoral area. A list of all Dalit eligible mothers of 6*2 VDC = 12VDCs was prepared with the help of local public health facilities’ community-based newborn program records, and female community health volunteers. More than 95% of all births were covered in their records and thus, our lists have included to a higher level of inclusiveness from the selected communities. Of the 348 eligible mothers, 15 women had migrated out of the district; therefore, 328 participants were interviewed.

### Instrument and data collection

The questionnaire was adapted from the Nepal Demographic and Health Survey (NDHS) 2011 [6].Observationchecklist was prepared to observe assets and animals in the house based on the NDHS 2011. A few minor words were changed in the original questionnaire to adapt the local context and increase readability of the questions. The face-to-face interview was conducted in the local language at respondent's house to collect information by two trained female interviewers who were trained by the first author and also involved in pretesting of questionnaires.

### Ethics

Ethics approval was obtained from the Institutional Ethical Review Board, Institute of Medicine, Tribhuvan University Nepal (Approval Number 79(6-11-E)^2^071/072. Informed written consent was also obtained from the district health office Mahottari and participants of the study. Personal identifiers were removed before the analysis of data and data were only presented as aggregate.

### Variables

The outcome variable was ‘place of delivery’ and delivery in health facility coded as ‘1’ and delivery in the home as ‘0’. Delivery in health facility includes delivery in either public (birthing centre, primary health care centre, district hospital, zonal hospital) or private sector (private hospital, NGO run hospital) [15]. The independent variables were selected based on comprehensive literature review and grouped into four major groups: socio-cultural, economic accessibility, physical accessibility and perceived need. Socio-cultural factors included ethnicity, mother’s education, husband's education, mother’s autonomy, and mother's current age, traditional healers, family type and family size. Economic accessibility factors were wealth status, mother’s occupation, husband’s occupation. The physical accessibility factors were the distance (home to the nearest health facility that provides delivery service), availability of motorized transport and means of transport. The perceived need factors were antenatal care, perceived quality of ANC service, parity of mother, planning of last pregnancy, advice for health facility delivery, birth preparedness and complication readiness, exposure to maternal health message, affiliation in mother's group/network, absence of health workers in health facility, perception of health worker’s behavior, visit with female community health volunteer [16, 17].

The age of respondent as reported by the individual was recorded. It was categorized into four groups: 15–19, 20–24, 25–29 and 30 years or above for analysis purpose [18]. Educational status was recorded as no formal education, primary, secondary, and certificate and above [6]. However, for bivariate analysis, it was re-coded to educated and uneducated. Occupation of the mother was broadly categorized into a housewife, daily wage, agriculture and job; and for a husband, it was categorized into the daily wage, agriculture, business and job[19]. Wealth status is a composite measure of socioeconomic status and adapted from NDHS 2011. It was derived using household assets and expenditures. Then the household wealth index was categorized into three groups poor to least poor corresponding from lowest to the highest status [6]. Woman’s autonomy included three components: control over finance, decision making power and extent to which she has the freedom to movement using 12 variables adapted from NDHS 2011. The women were categorized into three groups from low autonomy to high autonomy from the lowest to the highest. Ethnicity was adapted from further analysis of the 2011 NDHS. Mothers were asked whether they had heard about the birth preparedness; if yes, they were then prompted by reading the list of birth preparedness: saving money, arrangement of transport, finding blood donor, contacted with health worker to help in delivery, buy a safe delivery kit, arranged food & cloth[6]. The Dalit caste that was part of the study populations were ‘Terai Dalit’-Chamar, Mushahar, Dusadh/Paswan, Tatma, Khatabe, Banter, Dom, Chidimar, Dhobi, Halkhor and the ‘Hill Dalit’-Kami, Damai, Sarki, Gaine, Badi [9].

### Statistical analysis

Descriptive analysis was performed to report the rate of health facility delivery and characteristics of participants. The association of health facility delivery and other independent variables were first tested using Chi-square tests and the significant factors (p-value<0.05) were further entered for multi-variable analysis to ascertain the association of these factors with health facility delivery using backward LR methods [20]. Data analyses were performed using Statistical Package for Social Sciences Version 20.

## Results

### Characteristics of participants

Table 1 provides a description of the independent variables. The mean age of mother was 22.52 years (standard deviation 3.72). More than three-quarters of mothers (78%) and half of the husbands (58%) had no formal education. The majority of mothers (79%) were a housewife and 80% of husbands were engaged in daily wage work. Among the mothers who delivered in a health facility, half of them used an ambulance, followed by bull cart/tanga (24%) to reach a health facility. More than two third (71%) mothers did not found motorized transport easily at their residence village. The distance of health facility (that provide delivery service) from mother's house was more than 2 KM for 57% mother. The mean distance was 3.19 KM with SD 1.65.

**Table 1:** Description of socio-culture, economic, physical accessibility and perceived need factors.

Characteristics	Frequency (n = 328)	Percent
**Caste**		
Terai Dalit	296	90.2
Hill Dalit	32	9.8
**Mother age (Year)**		
15–19	68	20.7
20–24	159	48.5
25–29	79	24.1
≥30	22	6.7
**Family size**		
9 or more members	104	31.7
5–8 members	178	54.3
3–4 family members	46	14
**Type of Family**		
Joint or extended	226	68.9
Nuclear	102	31.1
**Mother's education**		
No formal education	257	78.4
Secondary	34	10.4
Primary	31	9.5
Certificate or above	6	1.8
**Husband's education**		
No formal education	192	58.5
Secondary	68	20.7
Primary	55	16.8
Certificate or above	13	4
**Practice of traditional healers**		
No	59	18.0
Yes	269	82.0
**Women Autonomy**		
Lowest	89	27.1
Middle	131	39.9
Highest	108	32.9
**Women's occupation**		
Housewife	259	79.3
Daily wage	62	18.9
Agriculture	3	0.9
Job	3	0.9
**Husband's occupation**		
Daily wage	263	80.2
Agriculture	21	6.4
Business	21	6.4
Mechanics	14	4.3
Job	9	2.7
**Wealth status**		
Lowest	109	33.2
Middle	109	33.2
Highest	110	33.5
**Means of transport during delivery (n = 98)**		
Ambulance	49	50
Bull cart/Tanga	23	23.5
Bus	14	14.3
Foot	9	9.2
Motorcycle	3	3.1
**Availability of motorized transport**		
No	233	71
Yes	95	29
**Distance (home to a health facility)**		
>2 Km	187	57
≤ 2 Km	141	43
**ANC visit frequency**		
<4 ANC	191	58.2
≥4 ANC	137	41.8
**Perceived good quality of ANC**		
No	264	80.5
Yes	64	19.5
**Parity of mother**		
3+	155	47.3
Second	93	28.4
First	80	24.4
**Planning of last pregnancy**		
No	185	56.4
Yes	143	43.6
**Advised for health facility delivery (n = 252)**		
No	113	44.8
Yes	139	52.2
**Birth preparedness and complication readiness**		
No	190	57.9
Yes	138	42.1
**Get transport incentive (n = 98)**		
No	31	31.6
Yes	67	68.4
**Association in women group/network**		
No	231	70.4
Yes	97	29.6
**Heard about the Safe Delivery Incentive Program**		
No	153	46.6
Yes	175	53.4
**Exposed to the maternal health message**		
No	236	72
Yes	92	28
**Absence of health workers in the health facility**		
No	227	69.2
Yes	101	30.8
**Perceived discriminatory behaviour of health worker**		
No	240	73.2
Yes	88	26.8
**Visit with female community health voluntary**		
No	70	21.3
Yes	258	78.7

### Health care related information about participants

The above Table 1 also shows that two in five mothers (42%) had visited four or more ANC visit while 58% of mothers had less than four. About one-fifth of mothers perceived ANC as good quality. Likewise about half (47%) of mothers were multiparous and more than half (56%) mothers had not planned their last pregnancy. As per the mother’s statement, mothers who visited any ANC, only 50% of them were suggested for health facility delivery. Similarly, more than half of the mothers (53%) mothers had heard about Save Delivery Incentive Program; however, only 68% mother had got transport incentive among who delivered in a health facility. In the same way, less than one third (28%) mothers had heard about the maternal health message through one or more media and 21% mothers were not visited by female community health volunteers during their last pregnancy. Similarly, 31% of mothers reported that they observed staff absence when they had visited health facility and 27% mother perceived staff’s behaviour as discriminatory in general while providing health services.

Table 2 presents birth preparedness and complication readiness and shows 58% of mothers had not done any preparation for birth at all. Among those mothers who had any preparation, 96% mother had saved money and 51% mother had arranged transport.

**Table 2:** Birth preparedness and complication readiness (N = 138).

*Responses	N	Percent
Saved money	132	96%
Arranged for transport	70	51%
Arranged for food	40	29%
Arranged for clothes	40	29%
Contacted health worker	26	19%
Bought safe delivery kit	6	4%
Found blood donor	2	2%

*Multiple responses

### Rates and factors associated with health facility delivery

Out of 328 interviewed mother, 70% mother had home delivery and only 30% (95% CI: 24.99, 34.81) mother had their childbirth in health facilities. It was found that the common reasons for not having a childbirth in a health facility were: not feeling necessary to go to health facility (63%), too far or lack of transportation (40%), cost related to service (40%), child born before reaching facility (8%), poor quality (1%), and husband did not allow (0.4%) to go health facility while giving birth to their baby.

Table 3 provides an association between health facility delivery and different factors at 95% CI in bivariate and multivariate analysis. Among socio-cultural, economic and physical accessibility factors, caste, mother’s age, family size, mother's education, father's education, women autonomy, mother's occupation, husband's occupation, wealth status, distance and availability of motorized transport were significant in bivariate analysis. Similarly, health-related significant factor in binary analysis were ANC visit frequency, perceived good quality of ANC, parity of mother, planning of last pregnancy, advice for health facility delivery, birth preparedness and complication readiness, association in women group/network, hearing about safe delivery incentive program, exposure to maternal health message, absence of health workers in health facility, perceived discriminatory behavior of health workers and visit with female community health volunteer. After adjusting for potential confounder, the regression analysis showed that ANC visits frequency (AOR: 3.54, CI: 1.82–6.90), birth preparedness and complication readiness (AOR: 3.15, CI: 1.60–6.17), planning of last pregnancy (AOR: 2.63, CI: 1.37–5.05), receiving advice from health workers during pregnancy (AOR: 3.96, CI: 1.99–7.86), and women autonomy (AOR: 2.24, CI: 1.03–4.89) were significantly associated with health facility delivery.

**Table 3:** Association of socio-cultural, economic, physical accessibility and perceived need factors with health facility delivery.

Characteristics	Crude OR (95% CI)	p-value	Adjusted OR (95% CI)	p-value
**Caste**				
Terai Dalit	1			
Hill Dalit	7.53(3.33–16.99)	<0.001	1.66 (0.33–8.16)	0.533
**Mother's age (Year)**				
≥ 20	1			
< 20	1.9 (1.09–3.31)	0.023	1.13 (0.41–3.11)	0.813
**Family size**				
≥6 member	1		1	
<6 members	1.88 (1.13–3.10)	0.014	2.36 (1.01–5.49)	0.047
**Family type**				
Joint or extended	1			
Nuclear	0.97 (0.58–1.61)	0.901		
**Mother's education**				
No	1			
Yes	4.43 (2.54–7.69)	<0.001	1.13 (0.48–2.63)	0.776
**Husband's education**				
No	1		1	
Yes	3.87 (2.35–6.36)	<0.001	1.82 (0.95–3.47)	0.068
**Women autonomy**				
1 (Lowest)	1		1	
2 (Middle)	2.77 (1.48–5.15)	0.001	2.25 (1.03–4.49)	0.042
3 (Highest)	1.25 (0.63–2.46)	0.519	1.20 (0.43–3.33)	0.722
**Health service from traditional healers**				
Yes	1			
No	1.38 (0.76–2.49)	0.291		
**Mother's occupation**				
Daily wage	1			
Non-daily wage	10.93 (3.33–35.80)	<0.001	1.45 (0.32–6.57)	0.628
**Husband's occupation**				
Daily wage	1			
Non-daily wage	2.89 (1.65–5.07)	<0.001	1.19 (0.51–2.81)	0.677
**Wealth status**				
1 (Lowest)	1			
2 (Middle)	1.82 (0.91–3.62)	0.088	1.92 (0.78–4.71)	0.152
3 (Highest)	6.02 (3.15–11.53)	<0.001	1.91 (0.78–4.67)	0.155
**Distance (home to health faculty that prove delivery service)**				
≥2 Km	1			
< 2 Km	4.87 (1.88–12.62)	0.001	1.88 (0.52–6.69)	0.329
**Availability of motorized transport**				
No	1			
Yes	2.50 (1.51–4.14)	<0.001	0.63 (0.26–1.55)	0.323
**ANC visit frequency**				
<4	1		1	
≥4	9.57 (5.48–16.71)	<0.001	3.54 (1.82–6.90)	<0.001
**Perceived good quality of ANC**				
No	1			
Yes	4.56 (2.57–8.08)	<0.001	1.18 (0.53–2.65)	0.676
**Parity of mother**				
Multiparous	1			
Primiparous	2.84 (1.67–4.79)	<0.001	1.69 (0.72–4.00)	0.226
**Planning of last pregnancy**				
No	1		1	
Yes	4.68 (2.81–7.80)	<0.001	2.63 (1.37–5.06)	0.004
**Advised for health facility delivery**				
No	1		1	
Yes	5.97 (3.29–10.83)	<0.001	3.96 (2.00–7.86)	<0.001
**Birth preparedness and complication readiness**				
No	1			
Yes	8.0 (4.64–13.77)	<0.001	3.15 (1.61–6.18)	0.001
**Association in women group/network**				
No	1			
Yes	2.09 (1.27–3.45)	0.004	0.70 (0.32–1.50)	0.367
**Heard about safe delivery incentive program**				
No	1			
Yes	3.94 (2.32–6.68)	<0.001	1.60 (0.81–3.18)	0.172
**Exposure to maternal health message**				
No	1			
Yes	3.31 (1.98–5.50)	<0.001	0.60 (0.28–1.28)	0.191
**Absence of health workers in health facility**				
No	2.74 (1.52–4.93)	0.001	1.21 (0.53–2.72)	0.641
Yes	1			
**Perceived discriminatory behaviour of health workers**				
Yes	1			
No	3 (1.72–5.23)	<0.001	1.46 (0.71–3.01)	0.295
**Visit with female community health volunteer**				
No	1			
Yes	4.15 (1.90–9.05)	<0.001	0.77 (0.25–2.33)	0.652

## Discussion

This is the first study that explores the rates of health facility delivery and their associated factors in the most disadvantaged Dalit community of Nepal. We found that the health facility delivery among Dalit was very low (30%), despite our setting being plain areas of Nepal where easier access to roads and transport is possible. The reasons for low health facility delivery were: mothers did not feel necessary, far distance, unavailability of transport and associated cost of institutional delivery. In the same way, few previous studies from Nepal and other countries have found similar reasons for not going for facility deliveries [6, 8, 14, 21]. Two important findings of this study were the association of the birth preparedness and attending four or more antenatal care visit to facilities delivery. Birth preparedness counselling and services are parts of antenatal care services in Nepal[14]. Such preparation gives mothers an opportunity to speak to health workers or health volunteers, be more prepared to take themselves to health facilities when necessary, and also a sense of connection with health facilities over the period of four or more visits and birth preparedness counselling. Studies from the Kaski district of Nepal, further analysis from NDHS 2011 and a study in South East Ethiopia showed that birth preparedness was a major determinant of institutional delivery [22–24]. Our findings, association of ANC visits with health facility are consistent with previous studies from rural Nepal, Ethiopia, Kenya and Bangladesh [17–19, 24–28].It has also been hypothesized that attending ANC visits enables mothers to recognize their needs to deliver in health facility critically looking at the dangers that are posed in home deliveries.

Advice from health workers and health volunteers has always been crucial in health promotion and maternal health. Indeed it is closely related to having frequent antenatal visits and birth preparedness. We also found that when a mother got advice for obtaining help from skilled birth attendants on delivering in health facilities, she was more likely to deliver in a health facility [17]. As mentioned earlier, ANC is one of the platforms to provide such education. The female community health workers and community outreach clinics are other sources where mothers get advice in their own community. Our findings indeed provide the evidence that these community-level services are still crucial in Nepal’s setting [12].

Mother middle autonomy was significantly associated with health facility delivery whereas highest autonomy was not associated. The reason may be that almost all of the mothers of the highest autonomy were belonging to nuclear family and their husbands had moved to a foreign country or depended on daily wage for their livelihood. This situation makes the mother hard to get their companion to go to a health facility when they are in labour pain. In such a scenario, probably a newer construct of autonomy is needed when mothers are all left alone at home but are burdened with the double responsibility of herself and her partner. A finding of rural Nepal showed that women’s highest autonomy was the significant factors for health facility delivery in bivariate analysis but not in multivariate analysis[18]. A study from Ethiopia showed that health facility delivery was increased when the mother could decide on health care spending along with her husband [29]. However, further research might need to explore more reasons for such association.

The mother who has planned their last pregnancy was about three times more likely to have institutional delivery. The study found that wantedness at time of birth increases the odds of having a doctor at delivery by 30% in South Africa while there was no such association in Brazil [30]. In Kenya, the rates of home delivery are increased by 40% when pregnancy is either unwanted or not wanted at that time [31].

This study is one of the few studies which report from the plain terrain of southern Nepal and covering the entire district. To best of authors’ knowledge, this is the first study on the most vulnerable and disadvantaged ethnic groups of Nepal (Dalit).The findings from this study could be useful in designing focused programs for such disadvantaged community. Increasing incentive to transportation and providing all the costs of maternal health services, including medicines may help families to overcome financially associated under utilization. The current findings also indicate the need for a maternal health program that is more precisely directed to vulnerable communities instead of current blanket approach intervention. However, many limitations have to be considered while interpreting the findings from this study. This study included only one district; therefore, its generality to the entire country is limited. While the study setting and the population is unique, the cross-sectional nature of the study lacks a temporal relationship. Mothers may have been more likely to report socially desirable behaviours instead of actual practices related to maternal health due to the use of local enumerators. Further, we did not explore the roles of husbands and other family members, which may influence the mother to have delivery in a health facility.

## Conclusion

Despite having free delivery services and financial incentive for transportation, less than one third (30%) Dalit mothers had institutional delivery in Mahottari. The common reasons for non-institutional delivery were that mother did not feel necessary of institutional delivery, too far distance, lack of transportation and the associated cost to delivery service. Further, more than fifty percentages mother (58%) had no birth preparation and complication readiness at all. This study identified that birth preparedness, ANC visit frequency and planning of pregnancy were the most important factors associated with health facility delivery. Proper counselling to mother about institutional delivery is instrumental and can be provided during ANC visit or when mother visits a health facility. Public health programs should also include such components which improve the mother's autonomy within and around family and empower the mother to negotiate with husband and/or family to have planned pregnancy. Future studies should focus on exploring the innovative approach to increase such service uptake in highly disadvantaged groups.

## Supporting information

S1 File S1 File is the supporting data of findings. (SAV)
